# Intraoperative ureter identification with a novel fluorescent catheter

**DOI:** 10.1038/s41598-021-84121-z

**Published:** 2021-02-24

**Authors:** Manuel Barberio, Mahdi Al-Taher, Eric Felli, Anila Hoskere Ashoka, Jacques Marescaux, Andrey Klymchenko, Michele Diana

**Affiliations:** 1 Institute of Image-Guided Surgery, IHU-Strasbourg, 1, place de l’Hôpital, 67091 Strasbourg, France; 2 Research Institute Against Digestive Cancer, IRCAD, Strasbourg, France; 3 Ospedale Cardinale G. Panico, Tricase (LE), Italy; 4 Maastricht University Medical Center, Maastricht, The Netherlands; 5 Laboratoire de Bio-Imagerie et Pathologies, UMR 7021, CNRS, Université de Strasbourg, Strasbourg, France; 6 iCube Laboratory, Photonics for Health, Strasbourg, France

**Keywords:** Ureter, Translational research

## Abstract

Iatrogenic ureteral injuries (IUI) occur in 0.5–1.3% of cases during abdominal surgery. If not recognized intraoperatively, IUI increase morbidity/mortality. A universally accepted method to prevent IUI is lacking. Near-infrared fluorescent imaging (NIRF), penetrating deeper than normal light within the tissue, might be useful, therefore ureter visualization combining NIRF with special dyes (i.e. IRDye 800BK) is promising. Aim of this work is to evaluate the detection of ureters using stents coated with a novel biocompatible fluorescent material (NICE: near-infrared coating of equipment), during laparoscopy. female pigs underwent placement of NICE-coated stents (NS). NIRF was performed, and fluorescence intensity (FI) was computed. Successively, 0.15 mg/kg of IRDye 800BK was administered intravenously, and FI was computed at different timepoints. Ureter visualization using NS only was further assessed in a human cadaver. Both methods allowed in vivo ureter visualization, with equal FI. However, NS were constantly visible whereas IRDye 800BK allowed visualization exclusively during the ureteral peristaltic phases. In the human cadaver, NS provided excellent ureter visualization in its natural anatomical position. NS provided continuous ureteral visualization with similar FI as the IRDye 800BK, which exclusively allowed intermittent visualization, dependent on ureteral peristalsis. NS might prove useful to visualize ureters intraoperatively, potentially preventing IUI.

## Introduction

Inadvertent genitourinary tract injuries complicate 0.3–1.5% of all abdominal surgical procedures. Iatrogenic ureteral injuries (IUI), particularly if not recognized intraoperatively, induce consistent morbidity and mortality and increase healthcare expenses^1^.

Intraoperative ureteral detection can be challenging, due to the retroperitoneal position of the ureters (often covered with a thick peritoneal and adipose sheet) and to the proximity of a number of tubular structures similar in size (e.g. gonadal vessels). Additionally, the presence of tissue inflammation, cancers or previous surgeries or irradiation may further complicate a safe identification of the ureters^2,3^, leading to an increased risk of ureteral damage. This failure to identify the relevant anatomy is the leading factor of IUI^4^. More than 50% of IUI occur during gynecological procedures^5^, and 26% of them have been observed during general surgical operations^6^. The minimally invasive approach also increases the risk of IUI^6,7^ due to the lack of tactile feedback and to the extensive use of energy devices^2^, potentially leading to ureter devascularization, crushing, thermal injury or laceration^8^. Immediate intraoperative IUI recognition and repair have a positive impact on long-term outcomes^9^. However, 50–70% of IUI are missed intraoperatively requiring a further operative procedure^10^. In such cases, a raised incidence of renal failure, urinary fistulas and sepsis, with a 40% higher mortality has been reported^11^. A technology allowing to intraoperatively enhance the visualization of the ureters, potentially reducing the risks and/or favoring an immediate identification of injuries, would have a relevant clinical impact. In this respect, preoperative ureteral stent placement preceding abdominal surgery has been extensively explored^3^. However, although this marginally invasive procedure seems to be safe, its usefulness in reducing IUI remains controversial^3,12^.

Intraoperative near-infrared fluorescence imaging (NIRF) is becoming a popular modality in the context of image-guided surgery. NIRF is based on the intravenous administration of a fluorescent dye, which is imaged by means of near-infrared (NIR) adapted cameras. Since the fluorophores emit light within the NIR spectrum, which penetrates deeper than visible light through tissue (up to 1 cm, depending on the tissue type and the exact NIR wavelength^13^), NIRF has a great potential to enhance visualization of structures in comparison to conventional laparoscopic imaging. To visualize ureters non-invasively, renally excreted fluorophores are required^14^. Currently, only 2 dyes are approved for clinical use, namely indocyanine green (ICG) and methylene blue (MB). Given the exclusive hepatic clearance of ICG, it has been used for intraoperative ureter visualization, through cumbersome retrograde injection by means of ureteral stent or cystoscopy^15,16^. On the other hand, MB has a partial renal clearance and is subsequently present at high concentration within the urinary tract, following its intravenous injection. However, this dye has inefficient optical properties (low quantum yield, poor brightness, and poor tissue penetration) and it requires modified NIRF imaging systems, most of which are not available for commercial use yet^14^. For such reasons, a number of new dyes are currently developed^14^, and among these dyes, the IRDye 800BK (nerindocianine sodium) has shown promising experimental results^17–20^. Due to its primary renal clearance and its optical characteristics similar to ICG (maximum absorption at 774 nm and maximum emission at 790 nm), allowing imaging with commercially available NIR cameras, the IRDye 800BK has already been tested in human patients. Currently, two clinical trials investigating the safety and efficacy (NCT03387410) or dosing (NCT03106038) of the IRDye 800BK have been completed and the results are pending publication.

Our group described an ultrabright and stable biocompatible fluorescent coating, namely the near-infrared coating of equipment (NICE), based on engineered NIR cyanine dyes with a bulky hydrophobic counterion^21^. NICE can coat surgical devices of any material and since it displays a similar spectral range as ICG, it can be imaged using commercially available surgical NIR cameras. It has been successfully used to coat a number of medical devices such as magnetic anastomotic devices during laparo-endoscopic gastrojejunostomies^22^, endoscopic clips to achieve laparoscopic tumor identification^23^, and Foley catheters to highlight the urethra intraoperatively^24^.

In the current study, we used NICE-coated commercially available double-J ureteral stents and compared their performance to IRDye 800BK for the NIRF intraoperative detection of the ureters in a porcine model. In addition, the efficiency of NICE-coated catheters to visualize human ureters in their natural anatomical position was assessed during a human cadaveric experiment.

## Methods

### Animals

The present study is part of the ELIOS protocol (endoscopic luminescent imaging for oncology surgery), fully approved by the local Ethical Committee on Animal Experimentation (ICOMETH No. 38.2016.01.085), and by the French Ministry of Superior Education and Research (MESR) (APAFIS#8721-2017013010316298-v2).

Six adult female pigs (*Sus* *scrofa* *domesticus*, mean weight: 44.7 ± 7.97 kg) were used. Animals were managed according to the directives of the European Community Council (2010/63/EU) and ARRIVE guidelines^25^. Following an acclimatation period in our animal keeping facility, the animals received premedication by means of an intramuscular injection of Zolazepam and Tiletamine 10 mg/kg (Zoletil ND, Virbac, France). Anesthesia induction was achieved through an intravenous injection of Propofol 3 mg/kg (Propofol Lipuro ND, B Braun, France) together with rocuronium 0.8 mg/kg (Esmeron ND, MSD, France). After intubation, the pigs were mechanically ventilated throughout the experiment and were sedated via an inhalation of isoflurane 2–3% (Isoflurin ND, Axience, France). During the experiment, analgesia was ensured with intramuscular buprenorphine (Buprecare ND, Axience, France) 0.01 mg/kg. At the end of the experimental procedure, pigs were sacrificed under deep anesthesia (Isoflurane 5%) with a lethal intravenous application of Pentobarbital 40 mg/kg (Exagon ND, Axience, France).

### Dye

The IRDye 800BK (nerindocianine sodium) (LI-COR Inc., USA) was used in this protocol. Its chemical composition is the following: C44H52N2O16S5, with a molecular weight of 1113 g/mol and an average mass of 1025.213 Da. Its configuration allows for a partial renal and hepatic clearance, with a maximum absorption found at 774 nm and a maximum emission at 790 nm. However, since it is highly hydrophilic, it is primarily metabolized by the kidneys, allowing for a non-invasive intraoperative ureteral imaging.

The IRDye 800BK powder was diluted in a sterile phosphate-buffered saline (PBS) solution to a concentration of 1 mg/mL (as per the manufacturer’s instructions). Based on earlier studies^18,19,26^, which have achieved optimal ureteral visualization, the concentration of 0.15 mg/kg was chosen.

### NICE coating

The near-infrared fluorescent coating is composed of a NIR dye with a specially selected counterion and a biocompatible polymer. The dye is based on Cyanine-7.5 derivatives, exhibiting optical properties comparable to indocyanine green. Since Cy7.5 dyes are cationic, in our preparation, their small inorganic anion iodide has been substituted by a bulky hydrophobic counterion tetraphenyl borate (TPB). The fluorescent coating was finally formulated by dissolving the synthesized cyanine dye and the biocompatible polymer poly(methyl meth-acrylate) (PMMA) in acetonitrile solvent. The synthetic process and the properties of the coating material are described in detail elsewhere^21^.

### Surgical procedure and data acquisition

**Figure 1:**
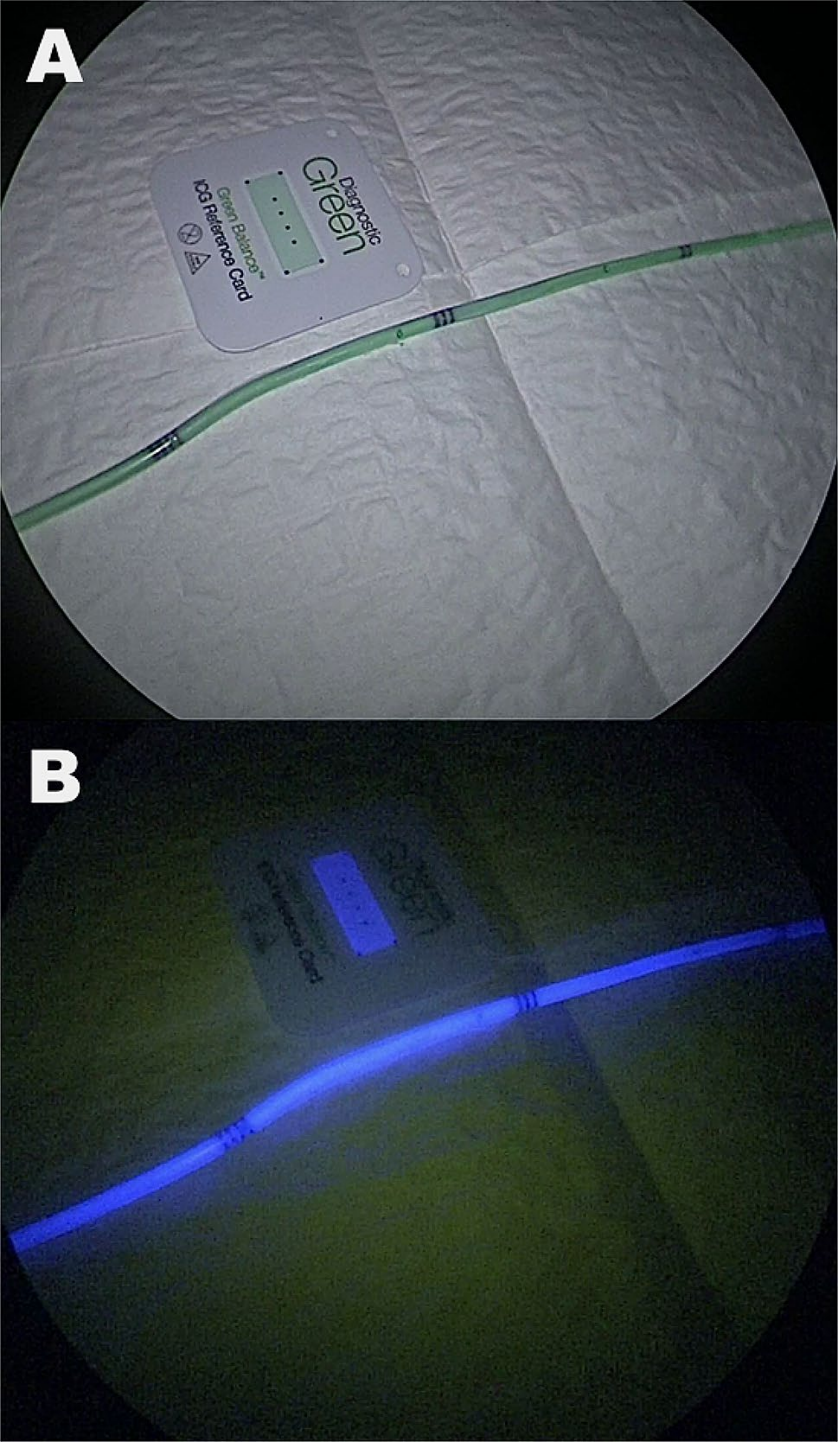
NICE-coated catheter demonstration under white light (**A**) and near-infrared light (**B**).

First, the retroperitoneum was surgically approached in an open fashion through a lateral subcostal incision. The proximal portion of the ureter was isolated and a ureterotomy was made. One pediatric double-J ureteral stent (3 French, 16 cm OptiSoft, OptiMed, Germany), which had been coated with 3 NICE layers one day prior to the experiment (Fig. 1) as earlier described^21–24^, was inserted into the ureter. The ureterotomy was closed using 6/0 PDS II (Ethicon, Johnson & Johnson, USA) and the retroperitoneal incision was closed in layers. Secondly, a laparoscopic procedure was performed using a 10 mm supraumbilical trocar and four additional 10 mm and 5 mm trocars. For laparoscopy, a commercially available NIR camera system (D-Light-P, KARL STORZ GmbH, Germany) was used. Following abdominal inspection, the ureter containing the coated double-J stent was imaged in NIR mode. Successively, a bolus of 0.15 mg/kg of IRDye 800BK was injected intravenously. The contralateral ureter (not containing the NICE-coated catheter) was imaged in NIR mode at 20, 40, 80, and 120 min. Every time a NIR video was acquired, a standard reference calibration card (Green balance ICG reference card, Diagnostic Green GmbH, Germany), emitting a constant fluorescent signal, was placed close to the target structure.

### Human cadaver experiment

For the ex vivo study, a human anatomical specimen was used. The human specimen was provided according to the standards of the American Association of Tissue Banks (AATB) and the requirements of the Uniform Anatomical Gift Act (UAGA) of the United States Government.

A 3-port laparoscopy (two 10 mm ports and one 5 mm port) was performed. The ureter was incised at the level of the pyelum and a NICE coated double-J ureteral stent (8 French, 30 cm, Opti-J, OptiMed, Germany) was inserted. A qualitative analysis of the ureteral visualization was then performed using the D-Light-P camera.

### Image and statistical analysis

The videos were analyzed postoperatively in order to compare the fluorescent signal during ureteral visualization with the two different means. An in-house software (ER-PERFUSION, IRCAD, France) was used to quantify fluorescence intensity. As previously described^19,27,28^, the fluorescent signal depends on the distance between the light source and the target structure, therefore in order to minimize this bias, normalized fluorescence (NF) was employed. NF was calculated by dividing the fluorescence intensity of the target area by the intensity of the reference card and it allowed to perform a comparative analysis regardless of the distance between the camera and the target organ. Every animal acted as its own control, since both interventions (NICE-coated catheter and IRDye 800BK) were performed in each animal.

Statistical analysis was performed using GraphPad 8.3 (GraphPad Software, USA). Statistically significance was considered with a p value < 0.05. A two-way ANOVA was performed to compare the NF of both interventions at each timepoint. A one-way ANOVA was used to compare the NF differences of the IRDye 800BK among the different timepoints.

## Results

### Animal experiment

**Figure 2:**
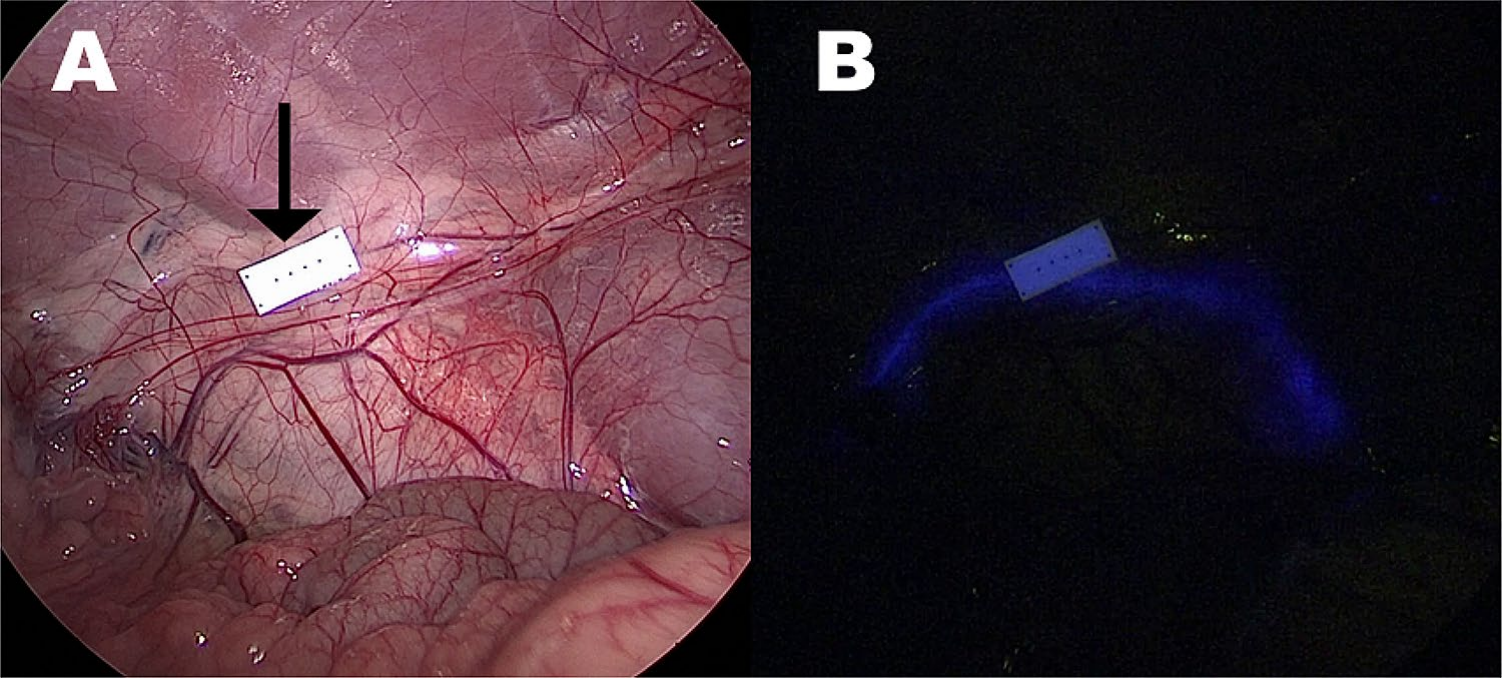
Demonstration of a NICE-coated catheter under white light (**A**) and near-infrared mode (**B**). The arrow highlights the fluorescence reference card. Differently from humans, the ureter is already visible under white light in pigs due to the thin overlying tissues.

**Figure 3:**
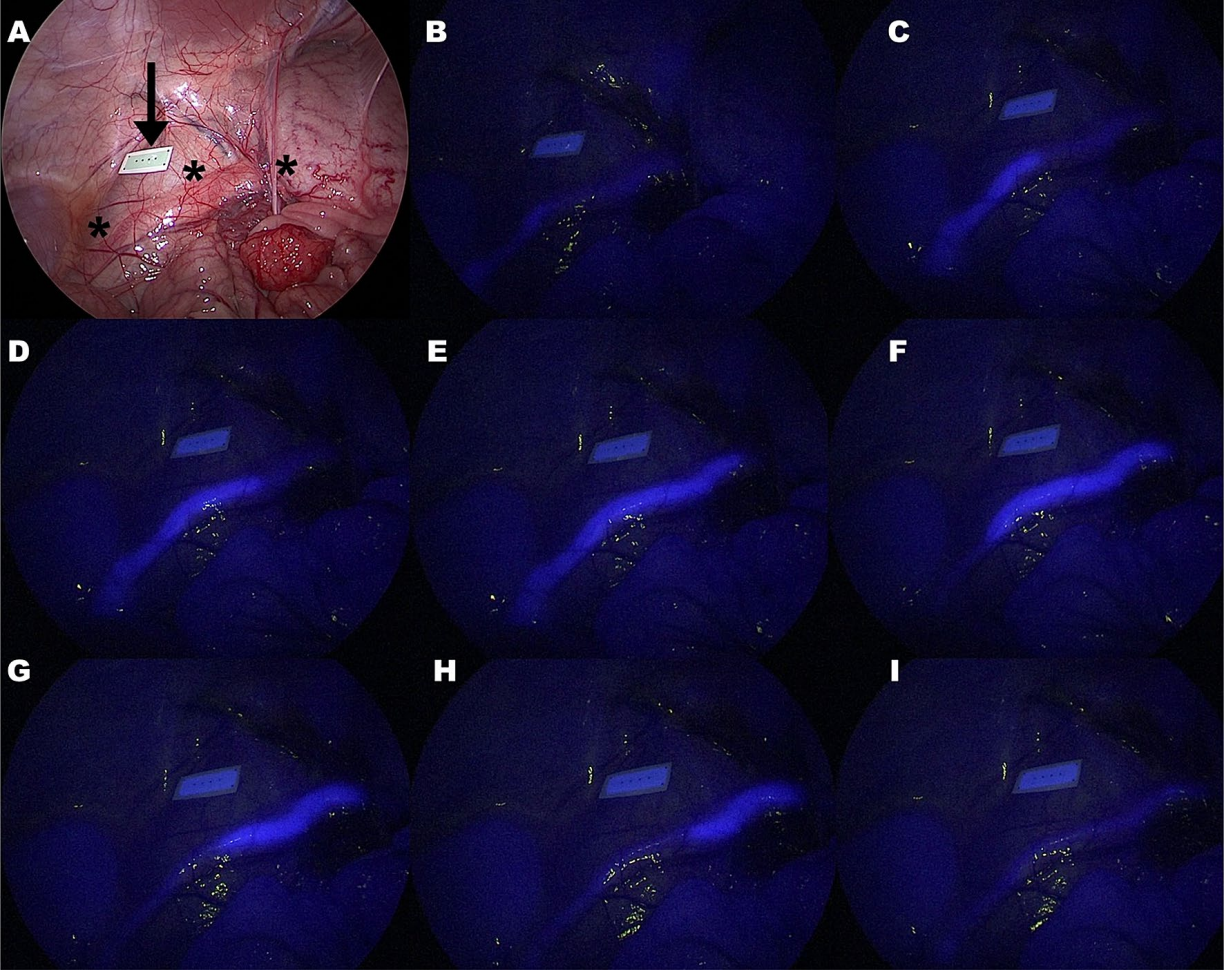
In (**A**), demonstration of the IRDye 800BK under white light. The stars indicate the ureter and the arrow the calibration reference card. In a near-infrared mode (**B**–**I**), the ureter is not clearly visible before (**B**) and after (**I**) a ureteral peristaltic wave (**C**–**H**) progresses. The duration of the peristaltic wave is variable, making the fluorescence-aided ureter visualization also variable. In this picture the time between photogram (**C**) and (**H**) was approximately of 50 s, whereas photogram (**E**), the only one showing the ureter entirely, was visible for 3 s, given the progression of the peristaltic wave. For this reason, using the dye the ureter was entirely visible only for 3 s, in this example. Additionally, the high background fluorescence given by the surrounding structures, which de facto limits clear ureter delineation, in absence of the peristaltic wave (**B** and **I**) is also visible.

The surgical procedures were performed without any complications or adverse events in every animal. Both the NICE-coated catheter and the IRDye 800BK allowed for a satisfactory visualization of the ureters under NIR light in each pig. However, the NICE-coated catheter was constantly clearly visible (Fig. 2) whereas the IRDye 800BK allowed for ureter visualization exclusively during ureteral peristaltic phases (Fig. 3), which occurred with irregular frequency in the animals.

**Figure 4:**
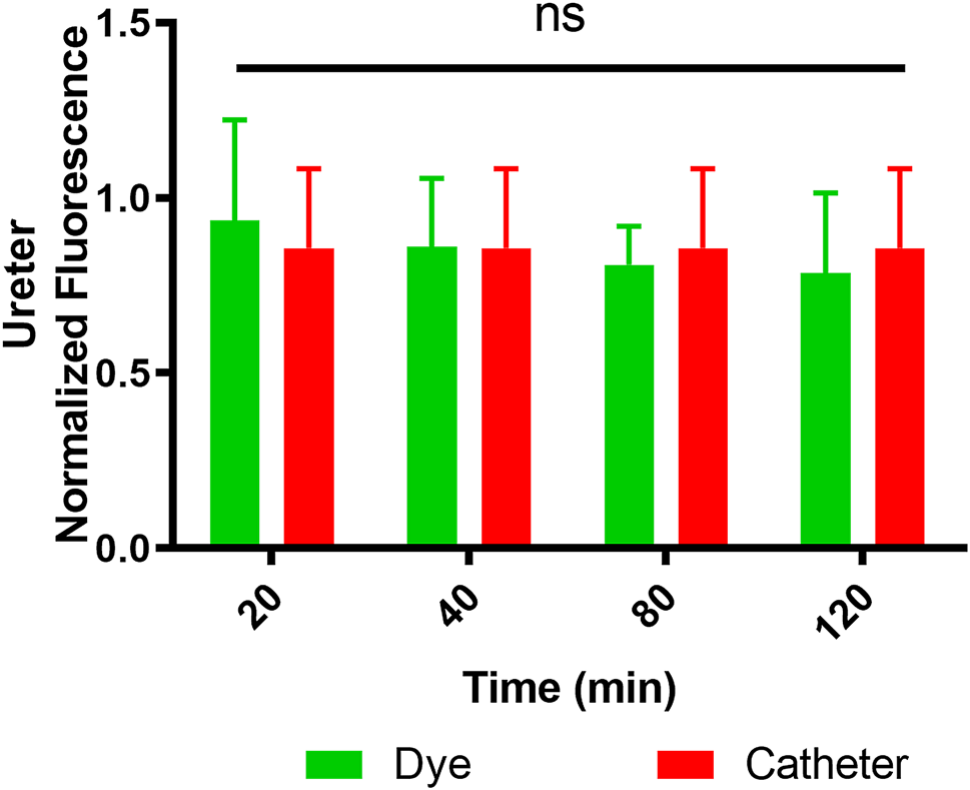
Graph showing the fluorescent signal intensity measured for the IRDye 800BK and the NICE-coated catheter in the porcine model. No statistical difference is present.

The NF of the IRDye 800BK computed during the ureteral peristalsis measured 0.94 ± 0.29 relative fluorescence units (RFU) at 20 min, 0.86 ± 0.19 RFU at 40 min, 0.81 ± 0.11 RFU at 80 min, and 0.79 ± 0.23 RFU at 120 min. There was no statistically significant variation in the dye’s NF intensity throughout the procedure. Additionally, there was no difference between the NF of the IRDye 800BK at any timepoint and the NF of the NICE-coated catheter, which measured 0.86 ± 0 RFU (Fig. 4).

### Human cadaver experiment

**Figure 5:**
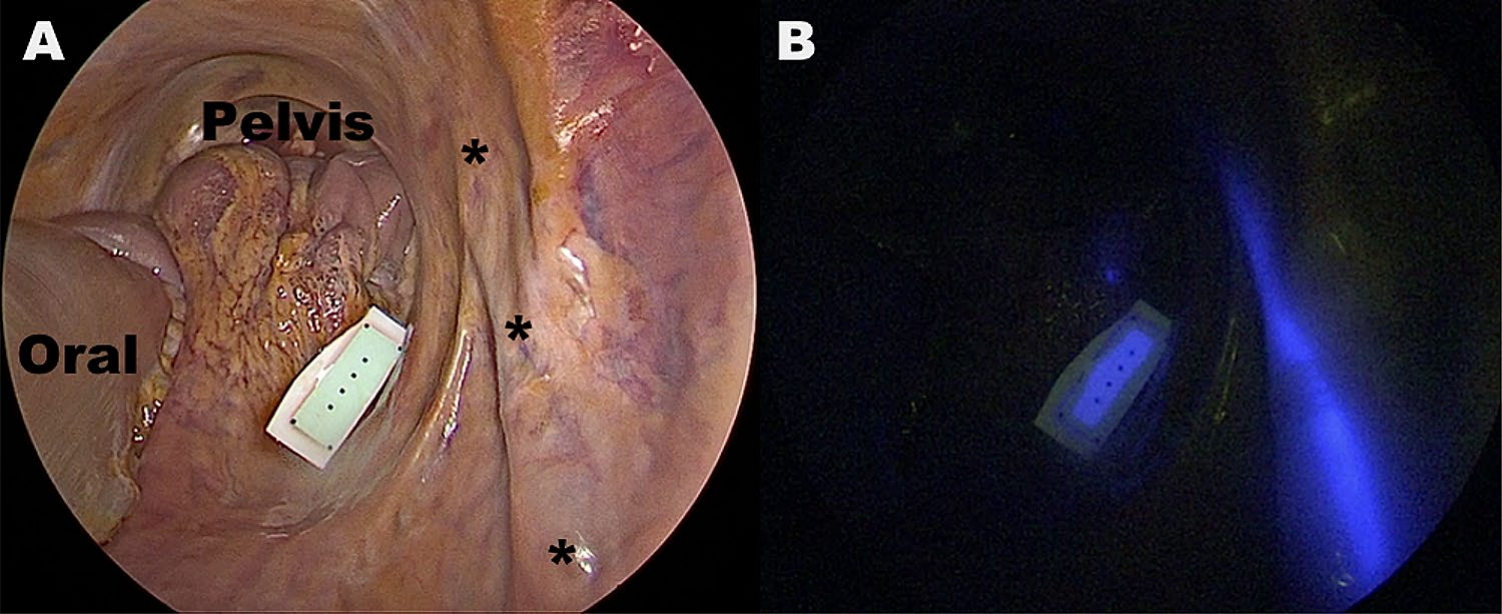
Demonstration of the efficiency of the NICE-coated catheter in detecting the undissected ureter under near-infrared light (**B**) in the human pelvis.

The NICE-coated catheter clearly highlighted the ureter in its natural retroperitoneal position, during NIR laparoscopy, as shown in Fig. 5.

## Discussion

Currently, there are no unitary prevention guidelines to avoid IUI^3^. As a matter of fact, while the European Association of Urology (EAU) recommends a cautious dissection with direct visualization of the ureters during abdominal/pelvic procedures^29^, the American Society of Colon and Rectal Surgeons (ASCRS) recommends the use of ureteral stents during complex diverticulitis cases^30^. Despite those recommendations, IUI still occur, representing a serious concern during abdominal surgical procedures. To minimize the risk of complications, in the era of precision surgery, the use of advanced optical platforms, allowing for an enhanced vision is mandatory, considering that operators exclusively rely on their optical perception. In this context, NIRF represents a promising solution due to its deep tissue penetration and its easy integrability within the minimally invasive surgical workflow.

In the current study, we obtained excellent ureteral visualization using a NICE-coated double-J catheter in the porcine model. Additionally, the fluorescence intensity of the NICE catheter was equal to the signal emitted by the preclinical IRDye 800BK, which has been explicitly engineered to ensure optimal NIRF ureteral enhancement. Yet, before a visible pulsatile urine flow occurs, IRDye 800BK highlights indistinctly every vascularized intra-abdominal structure, as a result of the initial intravascular phase following the intravenous dye injection. At this stage, the ureter might be confused with the neighbouring structures. However, as soon as the dye is excreted into the urine via peristaltic wave, the ureter becomes clearly enhanced and well distinguishable from the surrounding. This phenomenon became more evident over time, as the dye’s concentration decreased within the capillary system, while increasing within the pulsatile urine waves. During our experiments, we observed pauses > 1 min between peristaltic phases, which were increasing towards the end of the procedure. Differently from the IRDye 800BK, which highlighted the ureter only as long as ureteral peristalsis occurred, NICE-coated stents ensured a constant visualization of the ureter. Certainly, the IRDye 800BK allows for a totally non-invasive ureter visualization whereas the NICE-coated stent requires a cystoscopic placement. However, the performance of this promising dye has been described only in animal studies so far^18,19,26^. As pigs have less visceral adipose tissue than humans, their peritoneum is subsequently thinner. As a result, the ureters are often visible during white light laparoscopy without the need for any particular visual enhancement assistance. As a result, the ongoing human trials with the IRDye 800BK need to clarify if the dye is sufficiently performant to allow a satisfactory intraoperative identification of human ureters and if the intermittent fluorescence and high background fluorescence (given by the lack of exclusive urinary tract metabolization) observed during our study, allow for a satisfactory imaging. In the course of the human experiment, we were able to show that NICE-coated stents allowed for an optimal continuous visualization of the ureters in their native retroperitoneal position, without the need for any dissection, despite the thick peritoneal/adipose tissue sheet.

The preoperative placement of ureteral stents is reported to be safe^3^. However, this method harbors potential complications related to the ureteral catheter insertion and is associated with the costs (equipment and increased procedural time) resulting from the additional procedure.

Importantly, the controversy persists whether or not it prevents IUI from occurring^3^. The reason might be that conventional ureteral stents do not allow for a direct ureter visualization during laparoscopy, but rather, they require to be “felt” with the instruments. As a result, modified lighted ureteral stents, allowing for a diaphanoscopic ureteral visualization under white light in laparoscopic or robotic procedures, were introduced. Those devices have a high safety profile, since their placement is similar to normal ureteral stents and they showed promising results in retrospective trials^31,32^. However, they need to be wired to a dedicated external light source and they are consistently more expensive than non-lighted ureteral stents. On the contrary, NICE-coated catheters do not require any supplementary dedicated device. Therefore, one could imagine that once commercialized, their price would not differ greatly from the one of conventional stents. As previously demonstrated^21^, NICE is highly fluorescent (visibility > 0.5 cm depth within the tissue), perfectly biocompatible and non-toxic, without risk for any dye leakage and consequent cellular uptake. Additionally, NICE has proven exceptionally stable, since no alteration of the optical properties can be found even after 150 days in air and phosphate buffer medium, suggesting layer integrity of the coatings. For this reason, it is compatible with standard sterilization protocols, based on ethylene oxide or vapor, ensuring a potential expeditious clinical translation.

In this work, the use of NICE-coated commercially available double-J stents showed an optimal and continuous ureteral visualization in the porcine model. In addition, their efficiency in the human model was successfully assessed by using an anatomical specimen.

Drawbacks of this study lie in the small sample size and in the acute nature of the experiments. Additionally, in the porcine model, a surgical retroperitoneal approach was chosen for the placement of ureteral stents, since a cystoscopy in pigs is highly demanding due to anatomical reasons. A further limitation of our study lies in the comparison of the fluorescence signal of NICE-coated stents only assessed at one timepoint (before IRDye 800BK injection) versus the signal of the dye measured at different timepoints. We chose to collect the data in this manner, knowing that the NICE signal is highly stable (> 150 days)^21^ and with the intention to minimize a possible bias on the NICE fluorescence signal elicited by the circulating IRDye 800BK. Another limitation of our study lies in the fact that the fluorescently coated catheters were tested using thin, non-inflamed porcine and human tissue. Testing the performance of this technology within thickened inflamed tissue, even with preclinical models^33^, would be an interesting subject for future experiments, adding great value for the clinical translation.

The strong methodology which consists in using a large animal model together with a human anatomical specimen, as well as the promising results of this comparative study, are triggers for a prompt clinical translation of this technology. However, studies in human patients are required to understand the clinical usefulness of this technology.

## Conclusion

In conclusion, NICE-coated stents might represent a valuable innovation to allow for a continuous intraoperative NIRF ureter visualization, potentially preventing IUI from occurring.
